# Comparative transcriptome analysis of *Eriocheir japonica sinensis* response to environmental salinity

**DOI:** 10.1371/journal.pone.0203280

**Published:** 2018-09-07

**Authors:** Daizhen Zhang, Jun Liu, Tingting Qi, Baoming Ge, Qiuning Liu, Senhao Jiang, Huabin Zhang, Zhengfei Wang, Ge Ding, Boping Tang

**Affiliations:** 1 Jiangsu Key Laboratory for Bioresources of Saline Soils, Jiangsu Provincial Key Laboratory of Coastal Wetland Bioresources and Environmental Protection, Jiangsu Synthetic Innovation Center for Coastal Bio-agriculture, Yancheng Teachers University, Yancheng, China; 2 Key Laboratory of Biotechnology in Lianyungang Normal College, Lianyungang, China; 3 Chemical and Biological Engineering College, Yancheng Institute of Technology, Yancheng, China; Zhejiang University College of Life Sciences, CHINA

## Abstract

Chinese mitten crabs (*Eriocheir japonica sinensis*) are catadromous, spending most of their lives in fresh water, but moving to a mixed salt-fresh water environment for reproduction. The characteristics of this life history might imply a rapidly evolutionary transition model for adaptation to marine from freshwater habitats. In this study, transcriptome-wide identification and differential expression on Chinese mitten crab groups were analysed. Results showed: clean reads that were obtained totalled 93,833,096 (47,440,998 in Group EF, the reference, and 46,392,098 in Group ES, the experimental) and 14.08G (7.12G in Group EF 6.96G in Group ES); there were 11,667 unigenes (15.29%) annotated, and they were located to 230 known KEGG pathways in five major categories; in differential expression analysis, most of the top 20 up-regulated pathways were connected to the immune system, disease, and signal transduction, while most of the top 20 down-regulated pathways were related to the metabolism system; meanwhile, 8 representative osmoregulation-related genes (14-3-3 epsilon, Cu^2+^ transport ATPase, Na^+^/K^+^ ATPase, Ca^2+^ transporting ATPase, V-ATPase subunit A, Putative arsenite-translocating ATPase, and Cation transport ATPase, Na^+^/K^+^ symporter) showed up-regulation, and 1 osmoregulation-related gene (V-ATPase subunit H) showed down-regulation. V-ATPase subunit H was very sensitive to the transition of habitats. These results were consistent with the tests of qRT-PCR. The present study has provided a foundation to further understand the molecular mechanism in response to salinity changing in water.

## Introduction

Crustaceans are one of the most abundant and diverse animal groups, and they occupy diverse habitats [1–4]. It is well-known that some species such as the portunid live their whole lives in high-salt marine habitats. Interestingly, in the late Cretaceous, a marine crustacean ancestor left the marine environment, transitioned to freshwater or terrestrial habitats, and then quickly occupied most of the world’s rivers, lakes, rivulets, etc [5]. Now, some of them have completely adapted to their new low-salt environment and live in freshwater habitats throughout their lives (e.g., Freshwater crabs), while some of them are more specialized, spending most of their lives in freshwater, but moving to a mixed salt-fresh water environment for reproduction and larval development [6]. For the latter, in the reproductive season, they migrate to brackish estuary water to complete their habitat-mixing-dependent reproduction. The characteristics of this life history might imply a rapidly evolutionary transition model from freshwater to marine.

Adaptation to low-salt concentrations encountered by marine crustaceans was one of the most important challenges in their evolutionary history of transition from a marine to a freshwater environment. They must control osmoregulation to ensure the dynamic balance of Na^+^-K^+^ inside and outside tissue cells [7–8]. Some shellfish species (e.g., *Mytilus edulis*, *Scrobicularia plana*, *Mercenaria mercenaria*) maintain the salinity balance by changing both the closing frequency of their double shell and the expression of transport enzymes [9]. To prevent unrestricted water diffusion, freshwater fishes expel excess water by producing a large amount of low-concentration urine through high-rate glomerular filtration, and reabsorb glucose and inorganic salt through proximal and distal tubules; the salt lost by urination will be regained through the gills and food [10]. In recent years, gene expression and regulation in several osmoregulation-related biomarkers (Na^+^/K^+^-ATP, 14-3-3, HSPs, NKCCla, Apo-14) were studied with the development of molecular techniques [11–14]. Among these, the Na^+^-K^+^-ATP enzyme was an important type of heterogenous transmembrane protease system involved in regulating the osmotic balance of aquatic crustaceans by improving enzyme activity and expression [13]. Generally, its molecular structure, expression, morphology and even salinity change regulation have been extensively studied [15–29]. However, osmoregulation in aquatic crustaceans is a complex process with multi-proteins and multi-molecules. Furthermore, how to regulate the salt balance to rapidly adapt to increasing ambient salinity water habitats from fresh water was also a mystery for crustaceans (e.g., Chinese mitten crabs). Although, it had been reported that plasma cortisol concentration and gill Na^+^, K^+^-ATPase activity increased after exposure to high ambient salinity may be hypo-osmoregulatory mechanisms for whitefish (*Coregonus lavaretus*) [30], the osmoregulatory mechanism is very much species-specific [31]. Osmoregulation by Chinese mitten crabs (*Eriocheir japonica sinensis*) during physiological adaptation has been studied extensively, and recently genes (*CA* (carbonic anhydrase), *CYP4C* (cytochrome P450 4C), *GDH* (glutamate dehydrogenase), and the *NHE* (Na^+^/H^+^ exchanger)) involved in osmoregulation had been also reported [31]. However, the genetic basis of osmoregulatory mechanisms in Chinese mitten crabs is still poorly understood.

Nowadays, analysis at the transcriptome level has become increasingly more popular to reveal molecular mechanisms of physiological change in organisms [32–40]. In this study, we first aimed to reveal several functional genes related to osmoregulation by transcriptome analysis through Illumina paired-end sequencing, assembly, annotation and expression of differential genes during the transition of freshwater and saline habitats. Then, we would provide more information about candidate genes associated with the osmoregulation response to salinity change in habitats of crustaceans.

## Materials and methods

### Ethics statement

There are no specific permits for crab collection in the selected locations. The sampling locations are not privately owned or natural protected areas. Crabs used for the experiments are not considered endangered or protected species, and their collection is legal in China.

### Animals collection and challenge experiments

In this study, Twenty-four adult individuals of *E*. *j*. *sinensis* with carapace length more than 55 mm [41] were collected from the aquatic market in September 2016 and acclimatized at 20°C for 72 hours. Then, they were divided into two groups for construction of the comparative transcriptome database: EF (12) and ES (12). Group EF was cultured in freshwater for 120 hours, while group ES was challenged by a continuous increase of their water salinity: 5‰ (24 hours), 10‰ (24 hours), 15‰ (24 hours), 20‰ (24 hours) and 25‰ (24 hours). Finally, the three posterior gill tissues of the two groups were collected and frozen in liquid nitrogen for later experiments [42–43].

### Library construction and Illumina sequencing

The total RNA was extracted from the gill mixture of every group using TRIzol^®^ reagent (Invitrogen Corp., Carlsbad, CA, USA) according to the manufacturer’s instructions. Quality of degradation and contamination was monitored on 1% agarose gels, and its purity was checked using the NanoPhotometer^®^ spectrophotometer (IMPLEN, CA, USA). Concentration was measured using Qubit^®^ RNA Assay Kit in Qubit^®^ 2.0 Fluorometer (Life Technologies, CA, USA); 1.5 μg RNA per sample was used as input material for the RNA preparations from gill samples. The integrity of RNA was assessed using the RNA Nano 6000 Assay Kit of the Agilent Bioanalyzer 2100 system (Agilent Technologies, CA, USA). Sequencing libraries were generated using NEBNext^®^ Ultra^™^ RNA Library Prep Kit for Illumina^®^ (NEB, USA) following the manufacturer’s recommendations and sequenced on an Illumina Hiseq 2500 platform with paired-end reads.

### De novo transcriptome assembly

Raw reads were processed through Perl scripts, and then, clean reads were obtained by removing reads containing adapter, reads containing ploy-N and low-quality reads from raw data. At the same time, parameters such as Q20 and the GC-content of the clean data were calculated. Transcriptome assembly was accomplished using the Trinity programme (http://trinityrnaseq.sourceforge.net/) [44]. We obtained the transcripts by hierarchical clustering through Corset [45]. Finally, the longest transcripts for each gene were selected as unigenes. The value of N50 was also presented in this study to preliminarily estimate assembly quality.

### Functional unigene annotation and classification

The functional unigenes were annotated based on Nr/Nt (NCBI non-redundant protein/ nucleotide sequences, http://www.ncbi.nlm.nih.gov/), Pfam (Protein family, https://pfam.sanger.ac.uk/), KOG/COG (Clusters of Orthologous Groups of proteins, https://www.ncbi.nlm.nih.gov/COG/), Swiss-Prot (a manually annotated and reviewed protein sequence database, http://www.ebi.ac.uk/uniprot), KO (KEGG Orthologue database, http://www.genome.jp/kegg/) and GO (Gene Ontology, http://www.geneontology.org/). To obtain significant annotations, alignments with Nr, Pfam, KOG/COG, Nt, Swiss-Prot, KO and GO were carried out with a cut-off E-value of 10^−5^. Further classification of functional unigenes was conducted mainly by Gene ontology (GO) terms using Blast2GO, COG and KEGG [46].

### Differentially expression analysis

The DESeq R package was used for differential expression analysis of two conditions/groups [47]. The *p* values were adjusted using the Benjamini and Hochberg’s approach for controlling the false discovery rate (FDR) [48]. Genes with an adjusted p-value <0.05 found by DESeq were assigned as differentially expressed. GOseq R packages were implemented in GO enrichment analysis of the differentially expressed genes (DEGs)-based Wallenius non-central hypergeometric distribution. In addition, the KOBAS [49] software was used to test the enrichment significance of DEGs in KEGG pathways [50].

### Expression analysis by quantitative real-time reverse transcription PCR (qRT-PCR)

Representative DEGs were selected for confirmation of RNA-Seq (Quantification) data by qRT-PCR using a SYBR Premix Ex Taq kit (Aidlab) according to the manufacturer’s instructions. The 18S rRNA gene was used as an internal reference. Primers for qPCR were designed using Primer Premier 5.0 software. Reaction mixtures (20 uL) contained 10 μL of 2×SYBR^®^ Premix Ex TaqTM buffer, 1 μL of forward and reverse primers, 1 μL of cDNA, and 7 μL of RNase-free H_2_O. The thermal profile for SYBR Green RT-PCR was 95°C for 2 minutes, followed by 40 cycles of 95°C for 15 seconds, 55–60°C for 15 seconds, and 72°C for 30 seconds. The relative gene expression was analysed using the comparative threshold cycle method [51].

## Results and discussion

### Transcriptome sequence and assembly

Two cDNA libraries were constructed using gills from the freshwater group (EF) and the seawater group (ES) to obtain the transcriptome expression profile. Approximately 99,296,168 (49,845,580 in EF and 49,450,588 in ES) paired-end raw reads with a Q20% higher than 94.82% with a GC% of 42.15% and 46.02% in EF and ES, respectively, were obtained; This outcome could indicate good sequencing quality [52]. In this research, ultimately, 93,833,096 (47,440,998 in EF and 46,392,098 in ES) and 14.08G (7.12G in EF and 6.96G in ES) clean reads were obtained (File A in S1 File). The de novo transcriptome assembly by Trinity showed the total length and number of transcripts were 98,905,725 base pairs (bp) and 147,596 bp, respectively. The maximal length of transcripts was 18,715 bp (average length 670 bp; N50:1169 bp). The total length and number of unigenes were 79,287,774 bp and 76,307 bp, respectively (File B in S1 File). The maximal length of unigene was 18,715 bp with an average length of 670 bp. The parameter N50 for unigenes in this research was 1668 bp, which was slightly higher than that in other research also performed on crustaceans [53–55]. In this research, assembled sequences ranging from 300 to 1000 bp in length were the most abundant of the total sequence. A total of 10027 unigenes exceeded 2000 bp. For transcripts, the most abundant were clustered into a group of fewer than 1000 bp in length (File C in S1 File). In total, the assembly results also indicated that the length distribution pattern and mean length of the unigenes were consistent with previous transcriptome studies using Illumina Hiseq platform [56–58].

### Unigene functional annotation and classification

#### Functional annotation

BLASTX was used to search the protein database, and 76,307 assembled unigene sequences were identified after transcriptome assembly (147,596). The results revealed that 23,349 (30.59%), 8,329 (10.91%), 11,667 (15.28%), 19,813 (25.96%), 24,721 (32.39%), 24,899 (32.63%), and 12,369 (16.2%) of unigenes matched with the annotated sequences in Nr, Nt, KO, Swiss-Prot, Pfam, GO and COG databases, respectively. Ultimately, 3,879 of these unigenes were annotated in all databases, which account for 5.08% of the total unigenes (File D in S1 File).

#### GO functional classification

**Fig 1:**
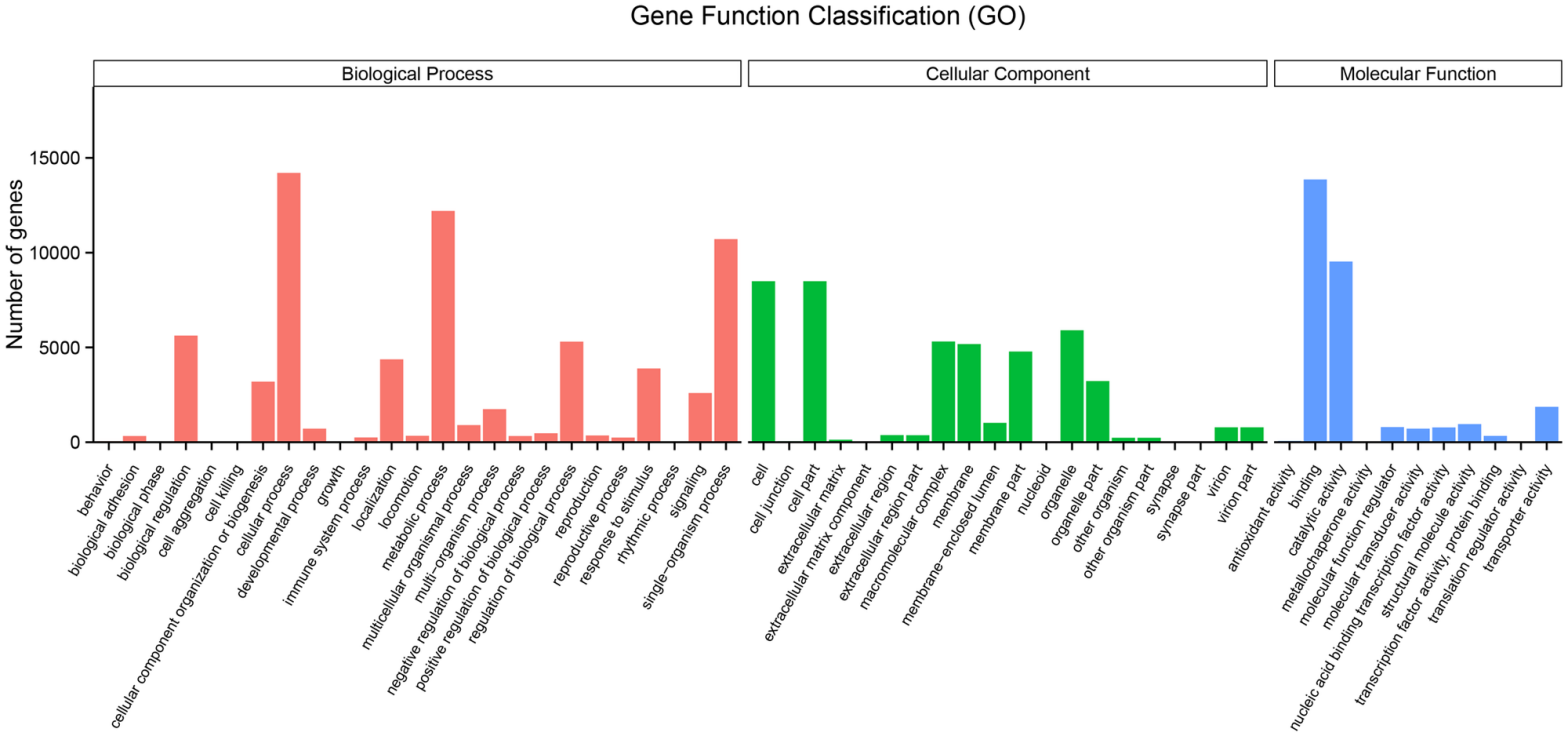
Gene ontology (GO) annotations of the assembled unigenes. 76,307 unigenes were classified into three GO categories containing 56 sub-categories.

GO classification is generally used to classify the functions of genes and their products in organisms. In this study, 24,899 unigenes were annotated in three main GO categories: cellular component (31.9%), biological process (47.8%) and molecular function (20.3%) (Fig 1). Three subcategories (cellular process, GO: 0009987; metabolic process, GO: 0008152; and single-organism process, GO: 0044699) of the category biological process and binding (GO: 0005488) of the category molecular function were identified in the transcriptome database with large numbers of unigenes (14,207, 13,863, 12,203 and 10,716, respectively). Within the cellular component category, 18 subtypes were annotated, and the most corresponding genes were involved in the cell part (8,488, 18.7%), the cell (8,488, 18.7%), the organelle (5,901, 13.0%), the macromolecular complex (5,309, 11.7%), the membrane (5,176, 11.4%), the membrane part (4,782, 10.5%), and the organelle part (3,223, 7.1%). The categories of the biological processes featured 25 subtypes. Most of the unigenes were assigned into cellular process (14,207, 20.9%), metabolic process (12,203, 18.0%), single-organism process (10,716, 15.8%), biological regulation (5,624, 8.3%) and regulation of biological process (5,303, 7.8%), localization (4,368, 6.4%), response to stimulus (3,886, 5.7%), cellular component organization or biogenesis (3,192, 4.7%), signalling (2,591, 3.8%), and multi-organism process (1,739, 2.6%) subcategories. In the molecular function category, most of the unigenes were included in binding (13,863, 47.9%) and catalytic activity (9,534, 33.0%).

#### COG functional classification

**Fig 2:**
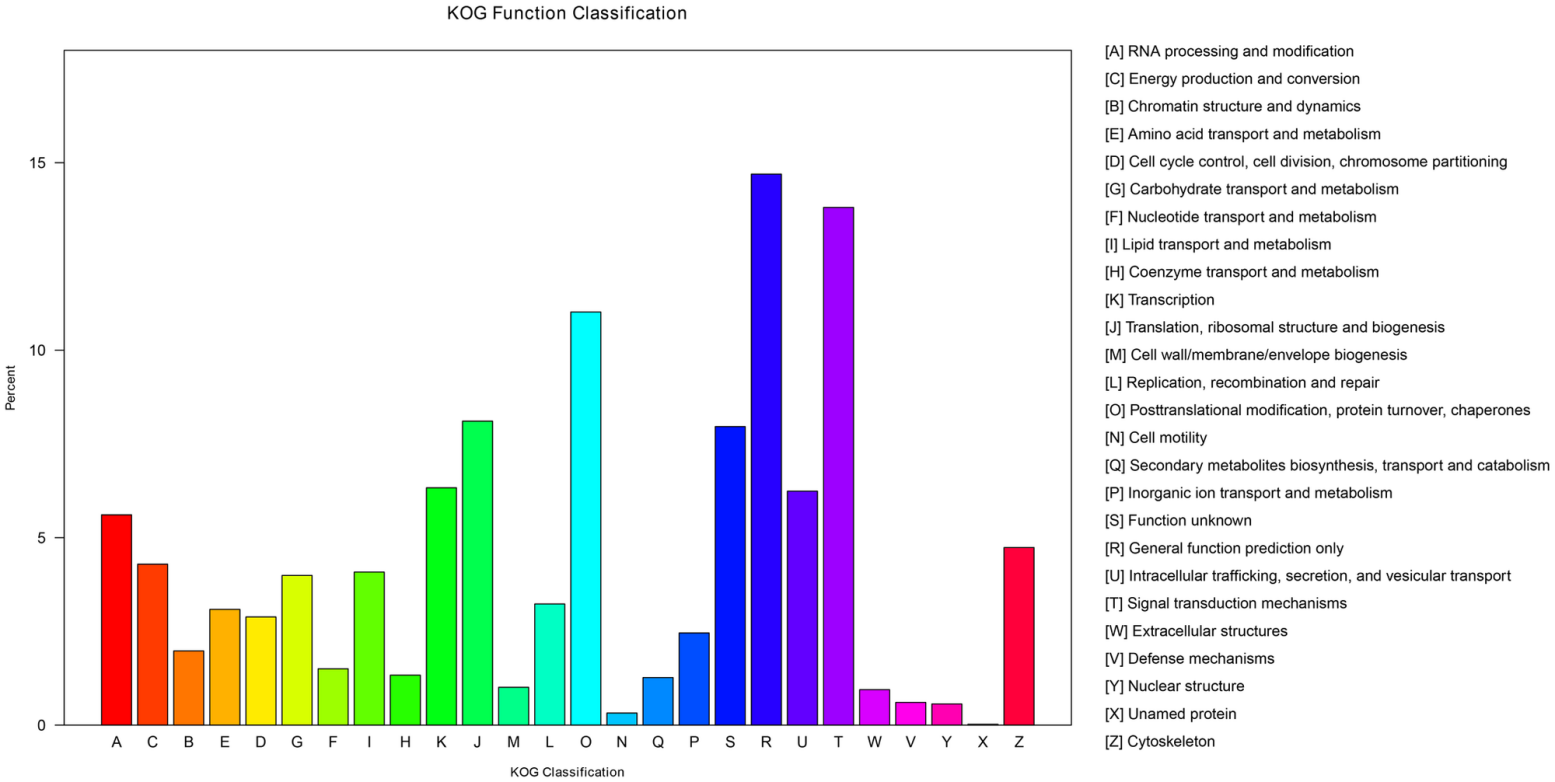
Cluster of Orthologous Groups annotations of assembled unigenes.

To evaluate the completeness of transcriptomes and the effectiveness of the annotation processes, the unigenes were also aligned to the COG database to phylogenetically classify the proteins. In the study, 12,369 (16.2%) unigenes were classified into 26 functional categories (Fig 2). The predominant categories were the cluster of general function prediction only (1818, 14.7%), signal transduction mechanisms (1708, 13.8%), posttranslational modification, protein turnover, and chaperones (1363, 11.0%), and translation, ribosomal structure and biogenesis (1003, 8.1%), followed by transcription (783, 6.3%), intracellular trafficking, secretion, and vesicular transport (772, 6.2%), RNA processing and modification (694, 5.6%), and energy production and conversion (531, 4.3%).

Transport and metabolism of ions should be important in osmoregulation, hence, unigenes that belong to the cluster of transport and metabolism were identified, including lipid transport and metabolism (505 unigenes), carbohydrate transport and metabolism (494 unigenes), and inorganic ion transport and metabolism (304 unigenes). Unigenes such as Na^+^/K^+^-ATPase, alpha subunit (Cluster-21379.0), multifunctional chaperone (14-3-3 family)(Cluster-173.0), aquaporin (major intrinsic protein family)(Cluster-18185.24311), Ca^2+^/Na^+^ exchanger NCX1 and related proteins (Cluster-18185.34230) might be involved in the molecular osmoregulation of aquatic crustacean. These results provide an important and valuable database for future research into the molecular osmoregulation mechanism of aquatic crustacean acclimated to different habitats on salinity.

#### KEGG functional classification

**Fig 3:**
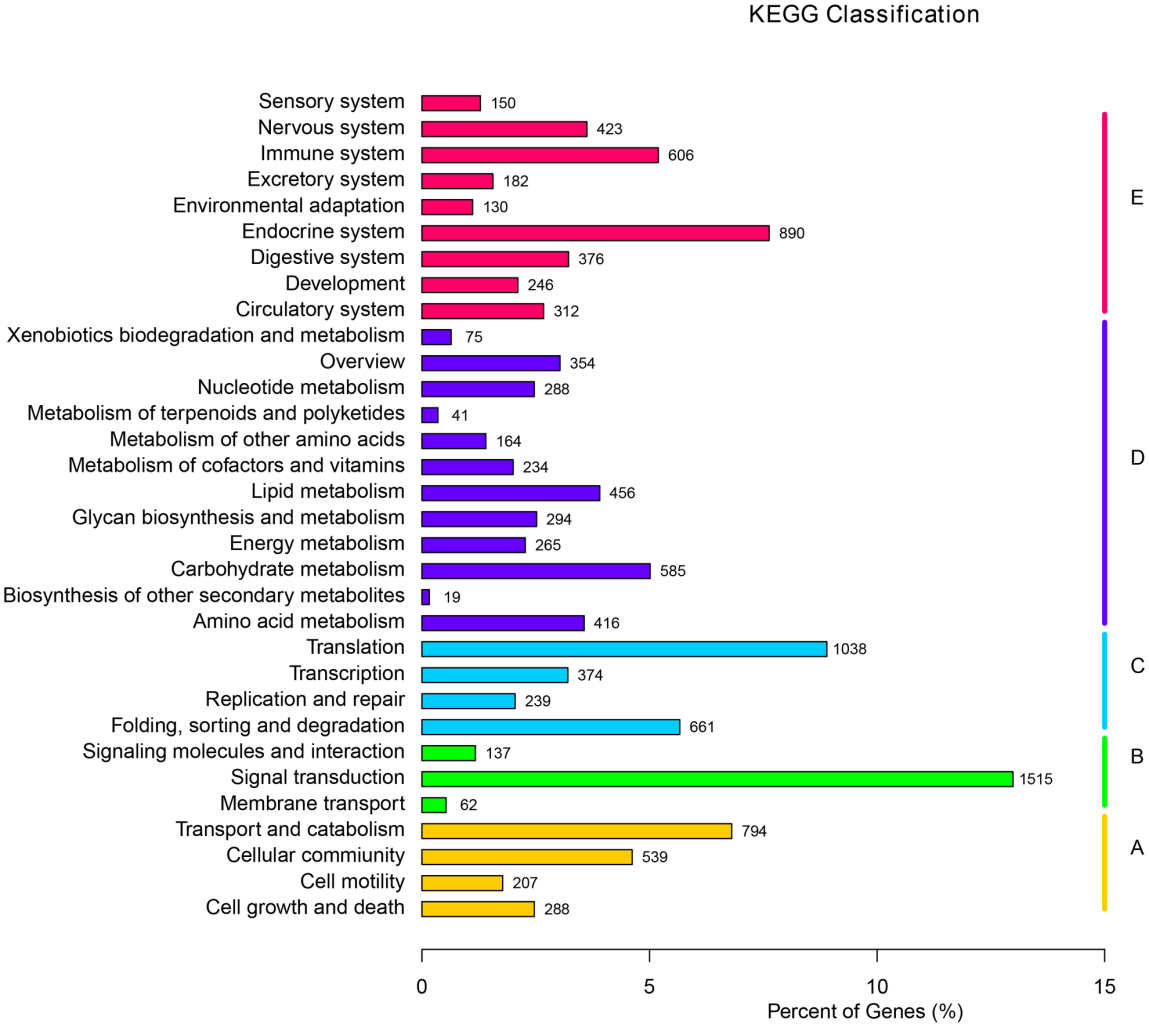
Cluster of KEGG Orthologue database annotations of assembled unigenes. A: Cellular Processes; B: Environmental Information Processing; C: Genetic Information Processing; D: Metabolism; E: Organismal Systems.

The unigenes were mapped to the reference pathways recorded in the KEGG database to understand the biological pathways of crustacean. A total of 11,667 unigenes (15.29%) were annotated in KEGG, located to 230 known KEGG pathways and classified into five major categories: cellular processes (1,828, 15.67%), environmental information processing (1,714, 14.69%), genetic information processing (2,312, 19.82%), metabolism (2,498, 21.41%), and organismal systems (3,315, 28.41%) with 32 subclasses in the KEGG database (Fig 3). Of the 230 predicted KEGG pathways, the organismal systems and metabolism pathways represented the two largest groups indicating that many organismal systems and metabolic activities during the habitat transition. Other pathways had large numbers of unigenes including pathways in ribosome (2.87%), endocytosis (1.58%), spliceosome (1.51%), protein processing in the endoplasmic reticulum (1.49%), oxidative phosphorylation (1.35%), and the PI3K-Akt signalling pathway (1.29%).

### Differential expression and enrichment analysis

In KEGG analysis, the top 20 pathways were significantly involved in response to salinity changes (*p*<0.05) (Table 1). Six pathways (30%) and a total of 102 unigenes were associated with metabolic processes (e.g., citrate cycle (TCA cycle), arachidonic acid metabolism, pancreatic secretion, glutathione metabolism, serotonergic synapse and nitrogen metabolism). Eight pathways (40%) and a total of 211 unigenes were associated with immune or disease processes (e.g., chemokine signalling pathway, B cell receptor signalling pathway, Fc gamma R-mediated phagocytosis, insulin signalling pathway, insulin resistance, platelet activation, shigellosis and bacterial invasion of epithelial cells). Thus, we have reason to believe that the unigenes in these pathways may play an important role in adapting to new environmental conditions during a habitat transition. Additionally, the top 20 up-regulated and top 20 down-regulated pathways of metabolic and immune systems were isolated. As a result, we found that most of the top 20 up-regulated pathways were related to the immune system, disease, and signal transduction, but nearly all the top 20 down-regulated pathways were related to metabolism systems (Table 2). We inferred that pathways related to the immune system, disease, signal transduction, and metabolism should be given more attention for revealing mechanisms of rapid adaptation during the transition from low-salinity freshwater to marine habitats. Studies in the short-term had partially revealed a relationship between metabolism and immunity that showed salinity (overnutrition) had impacts on crustaceans’ immune defence abilities with a significant increase in the activity of immune enzymes [29]. Metabolism and immunity, undoubtedly, are inextricably linked and coordinated by common regulatory axes [59].

**Table 1:** Significant enrichment analysis of differentially expressed genes in top 20 KEGG pathways.

Pathway	Gene number	Background number	Rich factor	*p*-Value	Function description
Focal adhesion	52	215	0.2419	0.0012	Cellular Process
Regulation of actin cytoskeleton	46	199	0.2312	0.0047	Cell motility
Citrate cycle (TCA cycle)	19	61	0.3115	0.0051	Carbohydrate metabolism
Chemokine signalling pathway	32	126	0.2540	0.0054	Immune system
Arachidonic acid metabolism	17	55	0.3091	0.0083	Lipid metabolism
B cell receptor signalling pathway	13	38	0.3421	0.0103	Immune system
Fc gamma R-mediated phagocytosis	22	87	0.2529	0.0193	Immune system
Insulin signalling pathway	38	174	0.2184	0.0193	Endocrine system
Insulin resistance	28	121	0.2314	0.0230	Disease
Platelet activation	33	150	0.2200	0.0256	Disease
Shigellosis	22	91	0.2418	0.0282	Disease
Pancreatic secretion	24	103	0.2330	0.0314	Digestive system
Hedgehog signalling pathway	15	56	0.2679	0.0331	Signal transduction
Glutathione metabolism	20	83	0.2410	0.0361	Metabolism
Dopaminergic synapse	24	105	0.2286	0.0370	Nervous system
Prolactin signalling pathway	13	47	0.2766	0.0377	Endocrine system
Bacterial invasion of epithelial cells	23	100	0.2300	0.0385	Disease
Serotonergic synapse	17	68	0.2500	0.0394	Digestive system
Nitrogen metabolism	5	11	0.4545	0.0443	Energy metabolism
AMPK signalling pathway	31	148	0.2095	0.0481	Signal transduction

**Table 2:** The profile of the top 20 up-regulated and down-regulated pathways in KEGG Orthologue database.

Type of regulation	Pathway	Rich factor	Adjusted *p*-value	Gene number	Function description
**Up-regulated**	Acute myeloid leukaemia	0.275	0.3628	11	Cancers
	Axon guidance	0.186	0.3936	29	Development
	B cell receptor signalling pathway	0.316	0.1962	12	Immune system
	Bacterial invasion of epithelial cells	0.230	0.1962	23	Infectious diseases
	Chemokine signalling pathway	0.222	0.1962	28	Immune system
	Dopaminergic synapse	0.210	0.3432	22	Nervous system
	Endocytosis	0.163	0.5257	46	Transport and catabolism
	ErbB signalling pathway	0.205	0.5257	16	Signal transduction
	Fc gamma R-mediated phagocytosis	0.241	0.1962	21	Immune system
	Focal adhesion	0.205	0.1935	44	Cellular Process
	Hedgehog signalling pathway	0.250	0.3432	14	Signal transduction Processing
	Hedgehog signalling pathway—fly	0.257	0.5257	9	Signal transduction
	Insulin signalling pathway	0.172	0.5257	30	Endocrine system
	PI3K-Akt signalling pathway	0.163	0.5257	38	Signal transduction
	Platelet activation	0.180	0.5257	27	Cancer
	Prolactin signalling pathway	0.255	0.3936	12	Endocrine system
	Proteoglycans in cancer	0.186	0.2138	42	Cancer
	Regulation of actin cytoskeleton	0.206	0.1935	41	Cellular Process
	Salmonella infection	0.221	0.2981	21	Disease
	Shigellosis	0.242	0.1962	22	Disease
**Down- regulated**	Adipocytokine signalling pathway	0.088	0.201	6	Endocrine system
	alpha-Linolenic acid metabolism	0.182	0.030	6	Lipid metabolism
	Arachidonic acid metabolism	0.182	0.002	10	Lipid metabolism
	Arginine and proline metabolism	0.123	0.026	9	Amino acid metabolism
	Ascorbate and aldarate metabolism	0.167	0.129	4	Carbohydrate metabolism
	Citrate cycle (TCA cycle)	0.098	0.160	6	Carbohydrate metabolism
	Ether lipid metabolism	0.122	0.085	6	Lipid metabolism
	Fat digestion and absorption	0.161	0.085	5	Digestive system
	Fatty acid degradation	0.084	0.173	7	Lipid metabolism
	Glutathione metabolism	0.096	0.085	8	Metabolism
	Glycolysis / Gluconeogenesis	0.069	0.160	10	Carbohydrate metabolism
	Linoleic acid metabolism	0.235	0.002	8	Lipid metabolism
	Pancreatic secretion	0.087	0.085	9	Digestive system
	Parkinson’s disease	0.052	0.302	13	Neurodegenerative diseases
	Protein digestion and absorption	0.094	0.224	5	Digestive system
	Pyruvate metabolism	0.080	0.201	7	Carbohydrate metabolism
	Starch and sucrose metabolism	0.087	0.201	6	Carbohydrate metabolism
	Steroid hormone biosynthesis	0.120	0.348	3	Lipid metabolism
	Valine, leucine and isoleucine degradation	0.094	0.085	8	Amino acid metabolism
	Vascular smooth muscle contraction	0.067	0.201	9	Circulatory system

### Osmoregulation-related genes

An interesting problem is discovering how osmoregulation-related genes are involved in ion transport and salt balance regulation in the transition from freshwater to saline water. According to the above results, most of the top 20 down-regulated pathways of Chinese mitten crabs were classified into metabolism pathways (some may relate to osmoregulation). However, most of the representative osmoregulation-related genes that encode the relative proteins (14-3-3 epsilon, Cu^2+^ transport ATPase, Na^+^/K^+^ ATPase, Ca^2+^ transporting ATPase, V-ATPase subunit A, Putative arsenite-translocating ATPase, Cation transport ATPase and Na^+^/K^+^ symporter) were up-regulated (*p*<0.05). Only the V-ATPase subunit H was down-regulated (*p*<0.05) (Table 3). The results of real-time RT-PCR showed that expression of DEGs annotated as Cluster-18185.13163 (14-3-3 epsilon), Cluster-18185.30584 (Cu^2+^ transport ATPase), Cluster-18185.15624 (Na^+^/K^+^ ATPase, beta subunit), Cluster-18185.21625 (Ca^2+^ transporting ATPase), Cluster-18185.25046 (Na^+^/K^+^ ATPase, alpha subunit), Cluster-18185.26540 (V-ATPase subunit A), Cluster-18185.27366 (V-ATPase subunit D), Cluster-18185.8525 (P-type ATPase), Cluster-18185.921 (Cl^-^ channel proteins) were up-regulated in treatment group compared to the control. Osmoregulation-related genes in the process of habitat transition included both up-regulation and down-regulation. These results were consistent with those of Hui et al. (2014), who performed a desalination-based transcriptome analysis on larvae of Chinese mitten crabs. Membrane permeability is related to osmoregulation [60]; for this reason, up-regulation of 14-3-3 epsilon protein may have an important role in osmoregulation because the 14-3-3 epsilon protein could suppress activation of Bcl-2, an antagonist of cell death, and further stop initiating mitochondrial membrane permeability [61]. Moreover, transporters are important osmoregulation factors responsible for the uptake and efflux of important substances such as inorganic ions, sugars and amino acids. This study also reflected that the Cu^2+^ transport ATPase, Na^+^/K^+^ ATPase, beta subunit, Ca^2+^ transporting ATPase, Cation transport ATPase, etc. were down-regulated in this study when adult Chinese mitten crabs were moved from fresh to saline water, as opposed to information in Hui et al. (2014) [37] about the V-ATPase subunit H (Cluster-18185.24276). This outcome implies sensitivity to ion flow regulation as part of V-ATPase to adaptation. Overall, this rapid adaptation to habitat transition must be related to many more pathways, such as environmental information processing, signal transduction, and energy metabolism.

**Table 3:** Representative osmoregulation-related genes differentially expressed.

Gene_ID	Description	Change	Log2 (ES/EF)	*p*-value
Cluster-18185.13163	14-3-3 epsilon	UP	1.4243	6.33E-13
Cluster-18185.30584	Cu^2+^ transporting ATPase	UP	4.7597	3.21E-13
Cluster-18185.15624	Na^+^/K^+^ ATPase, beta subunit	UP	6.1508	1.36E-45
Cluster-18185.21625	Ca^2+^ transporting ATPase	UP	2.6008	2.88E-09
Cluster-18185.24276	V-ATPase subunit H	DOWN	-1.2273	2.72E-46
Cluster-18185.25046	Na^+^/K^+^ ATPase, alpha subunit	UP	1.6011	0
Cluster-18185.26540	V-ATPase subunit A	UP	1.2083	5.99E-232
Cluster-18185.27366	V-ATPase subunit D	UP	1.0095	7.30E-24
Cluster-18185.30398	Putative arsenite-translocating ATPase	UP	1.5271	7.67E-05
Cluster-18185.14671	Cation transport ATPase	UP	4.9618	0.00016857
Cluster-18185.5348	Na^+^/K^+^ symporter	UP	4.1821	6.11E-06
Cluster-18185.8525	P-type ATPase	UP	2.3838	3.57E-07
Cluster-18185.921	Cl^-^ channel proteins	UP	5.0345	4.39E-06

**Fig 4:**
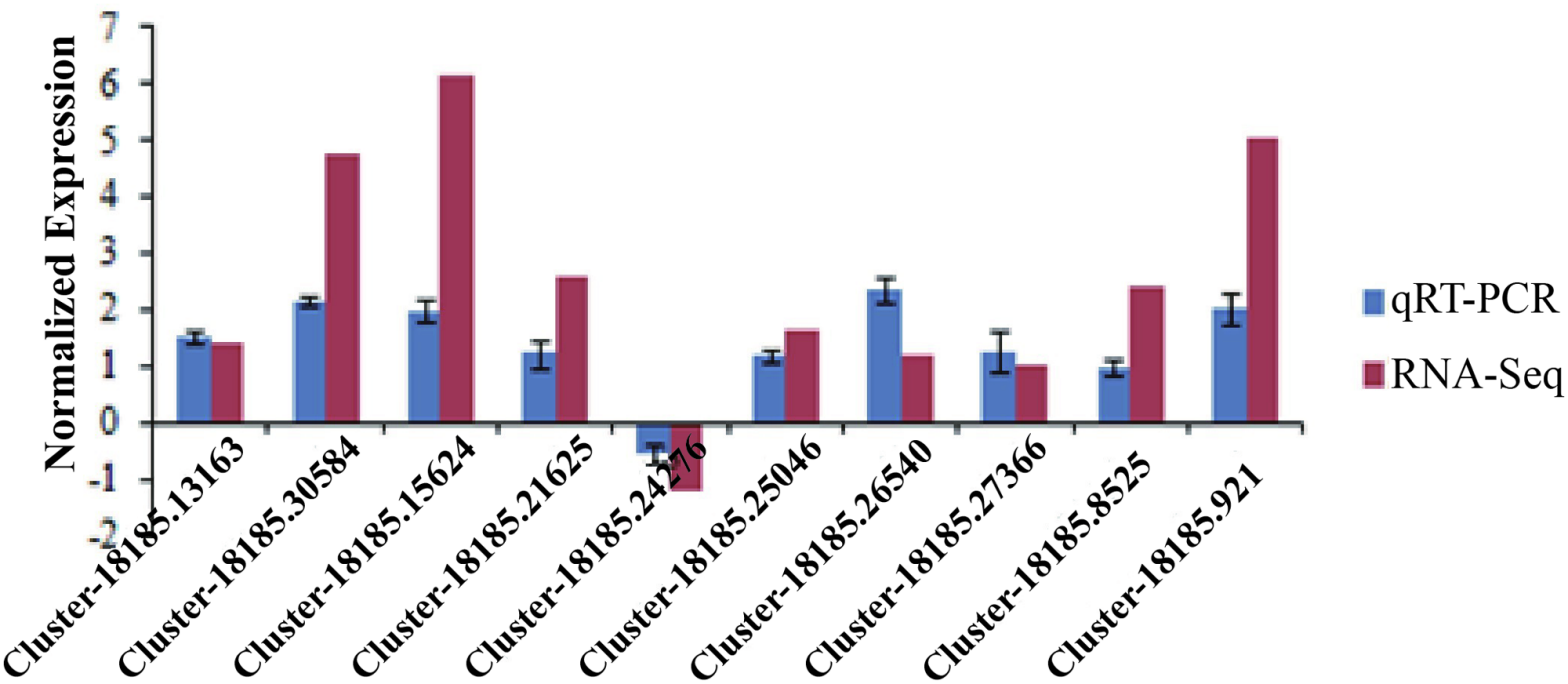
qRT-PCR analysis of representative differentially expressed genes. The expression of genes was normalized by logarithm. KO names or KOG descriptions of these genes are 14-3-3 protein epsilon (Cluster-18185.13163), copA, ATP7 (Cluster-18185.30584), ATP2B (Cluster-18185.21625), Na+/K+ ATPase, beta subunit (Cluster-18185.15624), ATPeV1H (Cluster-18185.24276), ATP1A (Cluster-18185.25046), ATPeV0A, ATP6N (Cluster-18185.26540), ATPeV0D, ATP6D (Cluster-18185.27366), DRS2, ATP8A (Cluster-18185.8525), CLCN2 (Cluster-18185.921).

In the late Cretaceous period, an ancestor of marine crustaceans left the long-term safety of the marine environment, transitioned to freshwater or terrestrial environments, and occupied most of the world’s rivers, lakes, and rivulets, etc. During their evolutionary history, crustacean ancestors regulated the balance of Na^+^-K^+^ inside and outside of the tissue cells by gene over-expression or mutation. In the present study, several functional genes related to osmoregulation have been revealed using transcriptome analysis and testing by qRT-PCR (Fig 4). We showed these genes might have played an important role in habitat transition of crustaceans. Perhaps, molecular adaptive evolution in the long term happened in some osmoregulation-related genes in the form of an increasing number of subunits, and these subunits then played an important role in rapid adaptation to the freshwater for Chinese mitten crabs. As transcriptome information from more crustacean species is accumulated, more detail in their rapid adaptation and molecular mechanisms will be available in the future.

## Supporting information

S1 File Statistics of transcriptomic profile on quality, assembly, length distribution and annotation. (RAR)
